# Supplementary material for: Faunal engineering stimulates landscape-scale accretion in southeastern US salt marshes
Source: Nat Commun. 2023 Feb 16;14:881. doi: 10.1038/s41467-023-36444-w (PMC9935860; doi:10.1038/s41467-023-36444-w)
Supplement: Supplementary file 1 — Supplementary Information [file 41467_2023_36444_MOESM1_ESM.pdf]

Supplementary Information

Supplementary Figure 1. Simplified conceptual model of salt marsh ecogeomorphology (after Fagherazzi et al. 2004[1]; 1). Features of marsh geomorphology (e.g., platform elevation and distance from the creek) structure inundation and salinity of marsh landscapes. Through exertion of effects on these physical stress-related variables, geomorphology structures key marsh ecology variables (e.g., vegetation biomass and zonation). However, the growth of this vegetation both above and belowground can alter tidal hydrodynamics, sediment deposition, and ultimately, marsh geomorphology. Therefore, models of salt marsh ecogeomorphology importantly consider the separate and combined, interactive effects of marsh (vegetative) ecology and geomorphology.

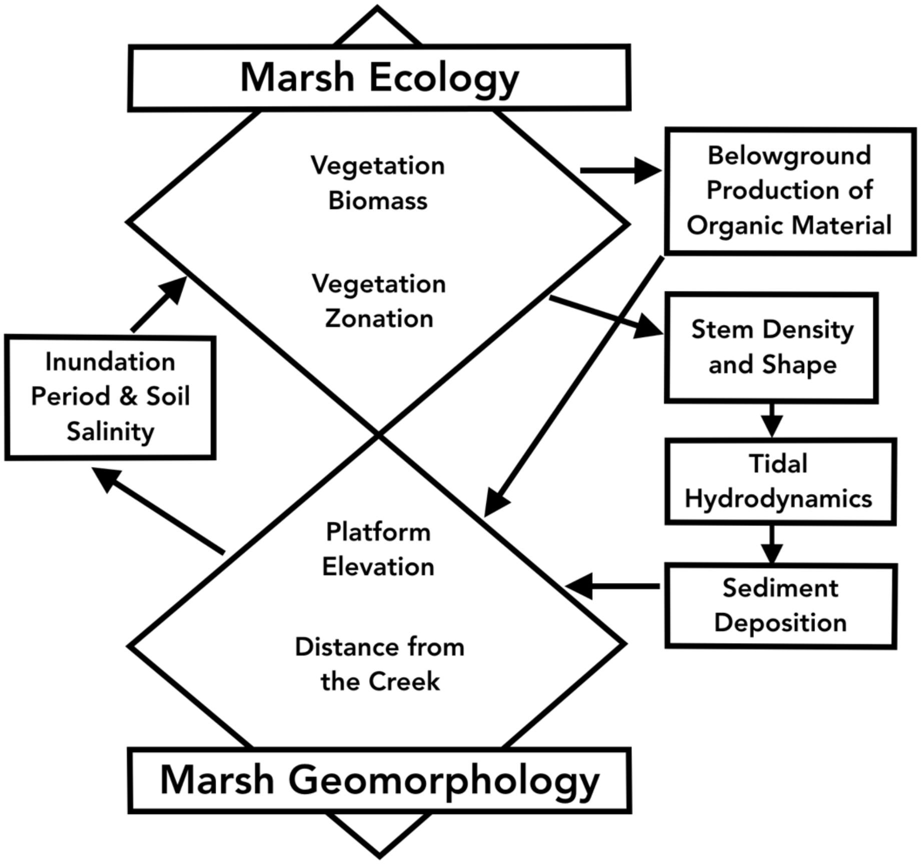

[1] S. Fagherazzi, M. Marani, and L. K. Blum. (2004). Introduction: The coupled evolution of geomorphological and ecosystem structures in salt marshes, in The Ecogeomorphology of Tidal Marshes, Coastal Estuarine Stud., vol. 59, edited by S. Fagherazzi, M. Marani,and L. K. Blum, pp. 1–5, AGU, Washington, D. C.

Copyright 2004 by the American Geophysical Union

Supplementary Figure 2. The hierarchy of geomorphology and species interactions that occur across the creekshed (top left), landscape (middle) and local patch (lower right) scales that predictably control the presence and population size of mussel populations in southeastern US salt marshes (figure from Crotty and Angelini 2020; 2). At the A) creekshed scale, longer tidal creeks with larger inlet cross-sectional areas support larger tidal prisms and higher creekshed recruitment and growth than shorter creeks of similar complexity. At the B) landscape scale, mussel aggregations in close proximity to the creek head are positioned at lower marsh platform elevations and experience higher water flows, recruitment, and growth than those located in the marsh interior. At the C) patch scale, mussels located on existing aggregations experience reduced predation and desiccation and have higher survivorship than mussels located off of existing mussel aggregations. The D) hierarchy of geomorphology and species interactions controlling mussel population recruitment, growth, and survival is organized by measured features, the physical/biological driver represented, and the mussel population effect (green coloring refers to creekshed scale, blue coloring refers to landscape scale, and yellow coloring refers to the patch scale).

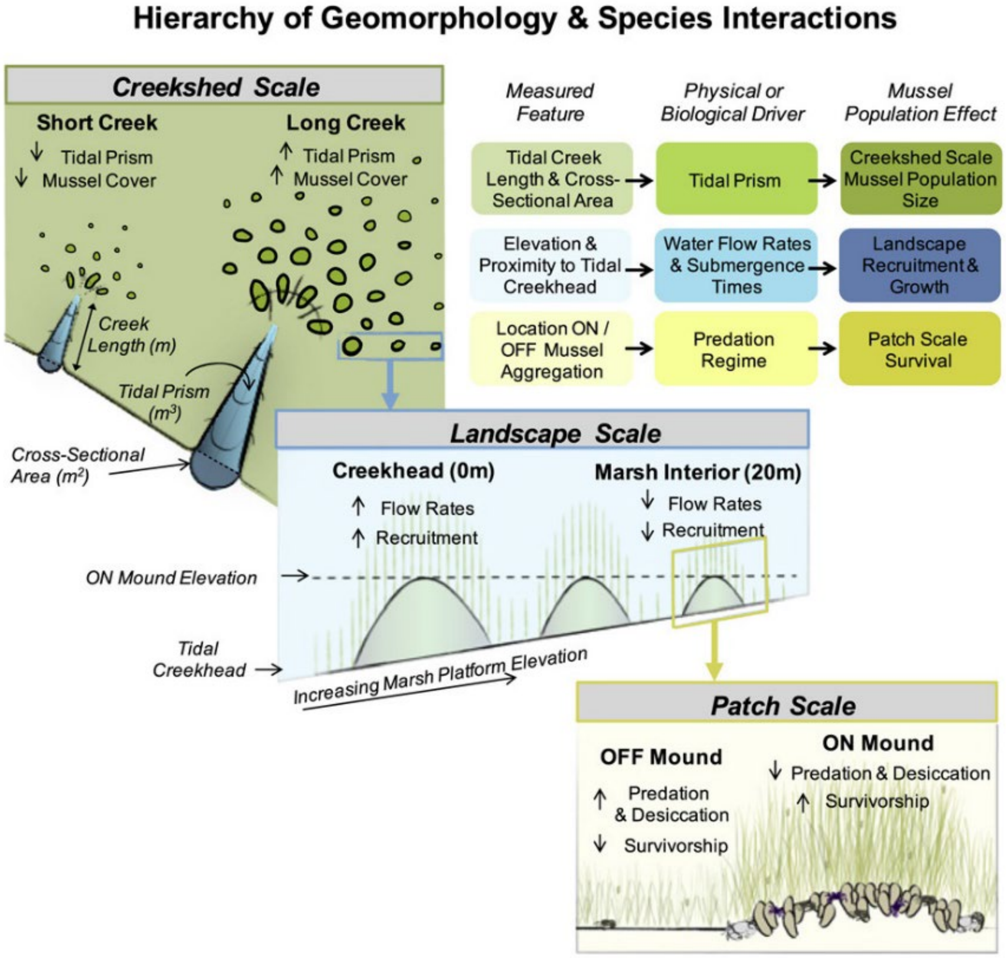

Supplementary Figure 3. Organic material results. (A) Average weight of surface organic material (colored bars) and inorganic material (grey bars) and (B) surface percent organic material (mean ± SE) collected on 4.7cm diameter filter papers during a 24-hour period of a summer spring tide. (C) Percent organic material from 5-cm cores (2.5cm diameter, mean ± SE). One-way ANOVA was used to determine if there was a significant main effect of marsh location on each of the response variables (organic and inorganic dry weight [$F_{4,38}$=9.5; p<0.0001; Adj. R^2=0.47], filter percent organic [$F_{4,38}$=9.0; p<0.0001; Adj. R^2=0.46], and core percent organic [NSD; p>0.20]). Letters denote statistically significant differences among treatments (Tukey HSD, p<0.001). No significant differences were recorded in the percent organic material of cores across marsh locations. N=8 filters or cores/location. All raw data points are presented as black circles in panels B and C.

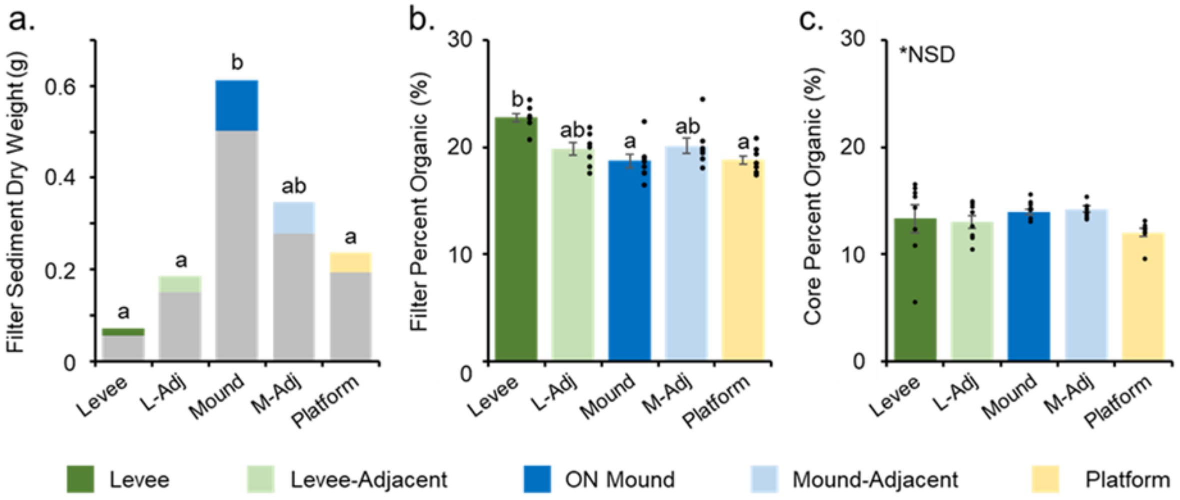

Supplementary Figure 4. Schematic of sediment catchment device. For experiment 2, sediment blocks were cut to 36cm x 36cm x 16cm and placed within plastic-encased bins of the same dimensions. Bins containing marsh blocks were then centrally placed and fitted within an additional larger bin (61cm x 61cm x 8cm), with the top of each box flush to the same height (see Side View, left). The outside bin was filled with 64, 5-cm diameter PVC poles and 32, 2.5-cm diameter PVC poles (both 8-cm in height) so that all bin edges were held upright and PVC was rigidly filling all space within the outer box (see Bird's Eye View, right. These sediment catchment units were then transported back to the experimental site where recipient holes were dug to the exact dimensions, so that the top of the marsh block (along with the top of each PVC pole) was exactly flush with the marsh surface sediment.

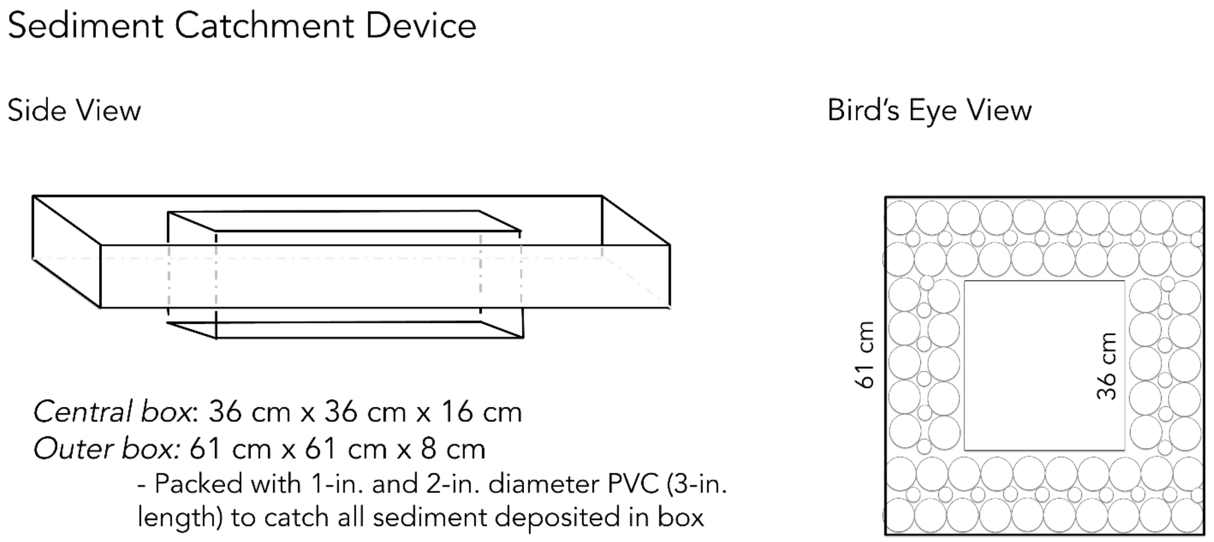

Supplementary Figure 5. Experimental design. Each sediment trap unit contained a central marsh transplant (36cm x 36cm x 16cm) of bare peat, peat with cordgrass, and/or peat with mussels. Surrounding the central marsh transplant was an outer box (61cm x 61cm with central cut-out, 8cm in depth) with 64, 5cm diameter PVC poles and 32, 2.5cm diameter PVC poles. The PVC reduced fetch and therefore trapped deposited sediment. The outer box was covered in 1cm-mesh hardware cloth to allow invertebrate access to marsh transplants. A) Treatments used in the marsh accretion experiment included: 1) open controls, 2) cordgrass controls, 3) singleton mussel plots, 4) small mounds (~20 individuals), 5,6) intermediate mounds both with and without cordgrass (50 individuals), and 7) large mound plots (80 individuals). Each treatment was replicated five times in each of (B) two experimental zones, the low elevation tidal creek head and the higher elevation marsh platform. (C) The field deployment (July 18- August 18, 2017) included elevated shades (pictured inset) above all treatments to minimize mussel mortality in the absence of cordgrass.

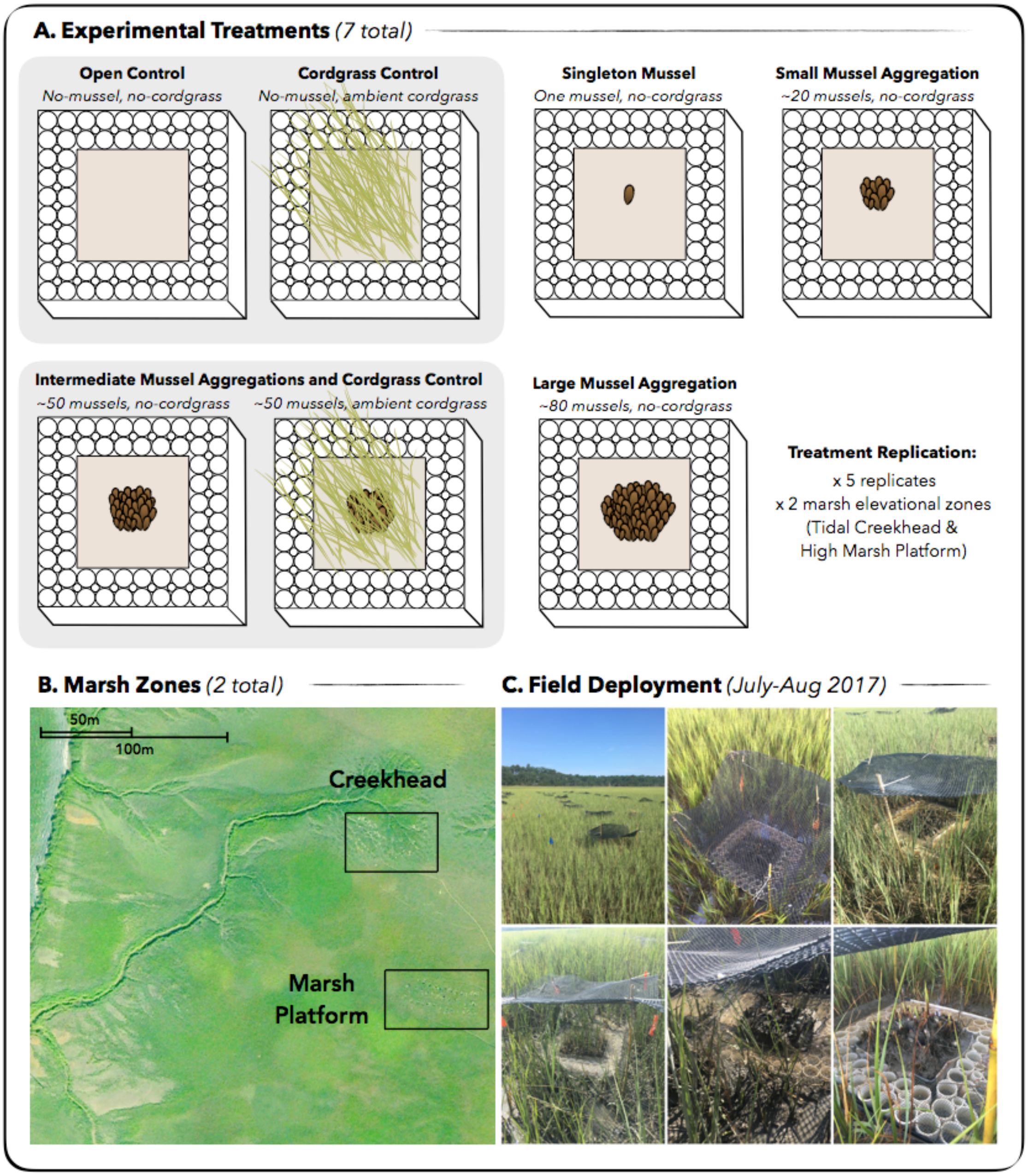

Supplementary Figure 6. Aboveground cordgrass biomass harvested at the completion of Experiment 2. All aboveground cordgrass biomass from the 36 x 36cm marsh block was cut at the marsh surface, cleaned, dried, and weighed at the end of the experiment (n = 5 plots/treatment/zone). Data are presented in box plots, including minimum, 25th percentile, median, 75th percentile, and maximum values as lines. Outliers are presented as solid circles. Aboveground cordgrass biomass (mean $\pm$ SD) was higher in all replicates that had mussel aggregations, both from the marsh platform (dark green, 91.7 $\pm$ 15.5 g / plot; n = 5) and the creekhead (light green, 89.2 $\pm$ 11.3 g / plot; n = 5). In plots without mussels, the aboveground cordgrass biomass was 54.8 $\pm$ 8.0 g / plot (n = 5) on the creekhead and 42.9 $\pm$ 7.0 g / plot (n = 5) on the marsh platform.

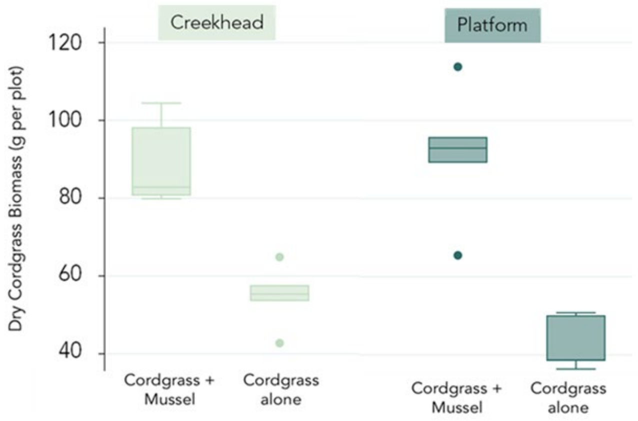

Supplementary Figure 7. Initial elevation data collection for the creekshed mussel manipulation. Dashed boxes indicate the boundary of the 2,500m^2 creekhead area. White diamonds indicate that an RTK elevation point was taken prior to experimental deployment, while solid lines indicate location of 50m^2 transects. Within each transect, each mussel aggregation was scored and its height was recorded. Marsh elevation was inferred by subtracting mound height from the mussel mound height ceiling calculated for the region.

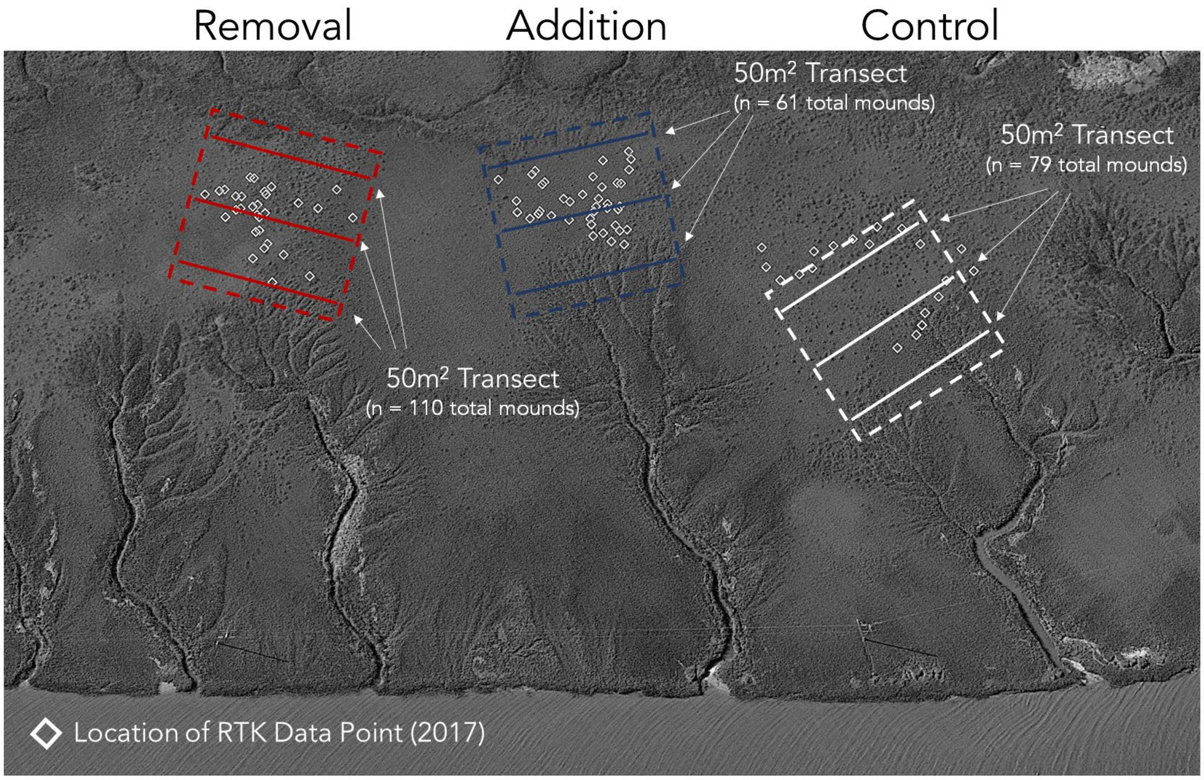

Supplementary Figure 8. Exponential fitting of the Total Suspended Sediment (TSS) concentration data surveyed at Dean Creek (blue dots), located in Sapelo Island, Georgia, USA. The time is in seconds from the reference time, which corresponds to the time of the first survey performed at slack water.

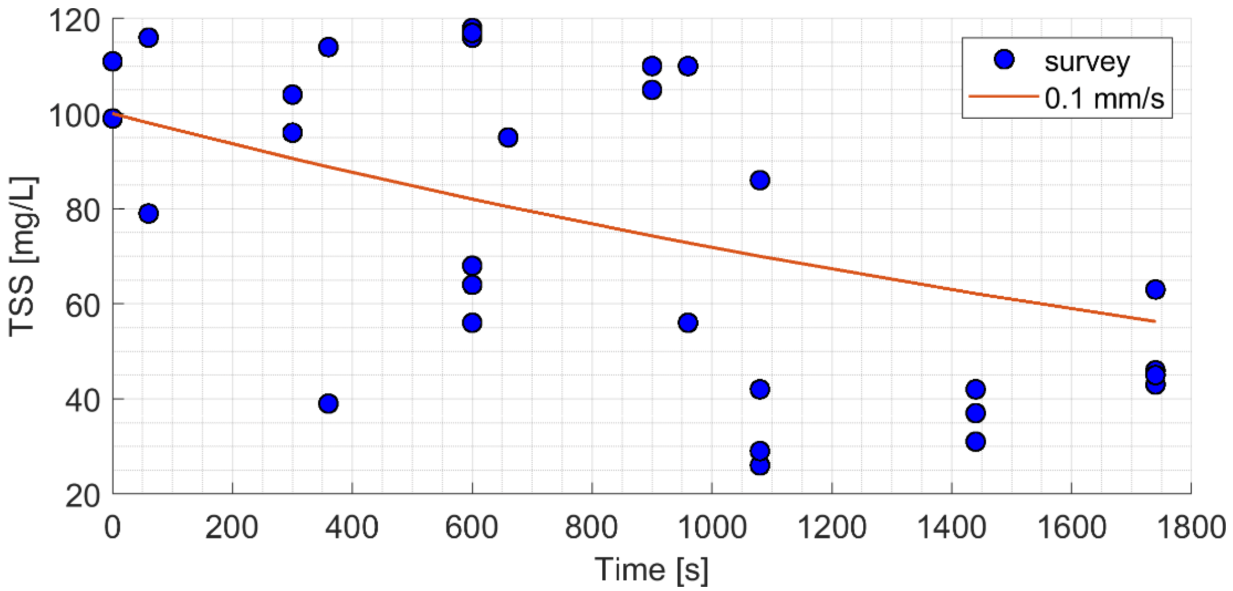

Supplementary Figure 9. Sediment deposition in the model domain, expressed in meters per year, compared to the mussel density used in the numerical simulations. The results refer to the scenario where mussel mounds occupy 20% of the creek head area. The intercept is set equal to the sediment deposition obtained where there are no mussels in the domain.

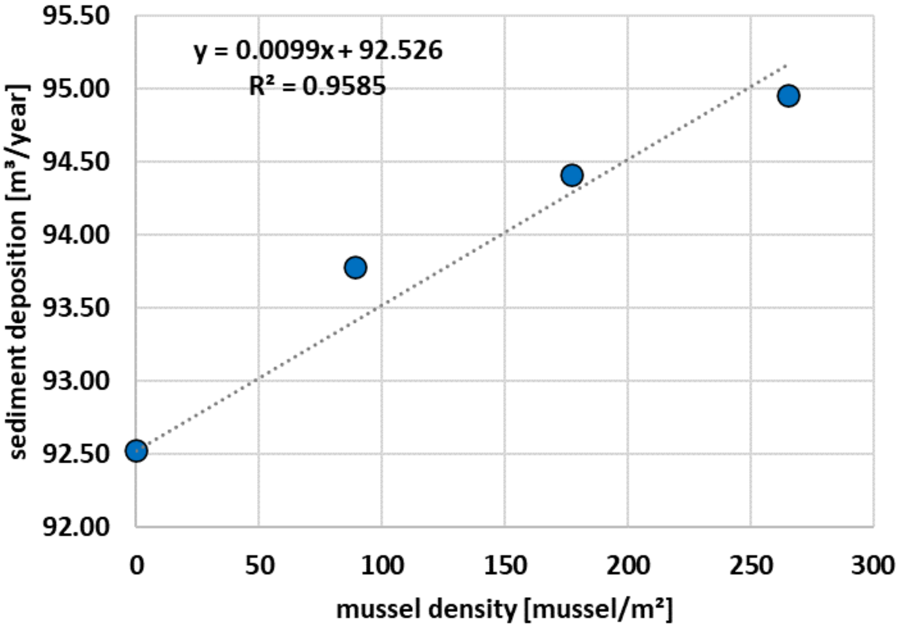

Supplementary Table 1. Mussel and cordgrass biomass effects on sediment deposition in the creekhead and the marsh platform zones.

Creekhead Model: $F_{(2,32)} = 58.7$; $p < 0.0001$; Adj. $R^2 = 0.77$

	Coefficient	*Std. Error*	*t-Statistic*	*Prob.*
Mussel Biomass (kg m^{-2})	0.013	0.001	10.7	p<0.001
Cordgrass Biomass (kg m^{-2})	0.033	0.031	1.1	p=0.289
Constant	0.076	0.012	6.4	p<0.001

Platform Model: $F_{(2,32)} = 53.4$; $p < 0.0001$; Adj. $R^2 = 0.75$

	Coefficient	*Std. Error*	*t-Statistic*	*Prob.*
Mussel Biomass (kg m^{-2})	0.006	0.0006	10.3	p<0.001
Cordgrass Biomass (kg m^{-2})	0.0001	0.014	0.01	p=0.993
Constant	0.063	0.006	10.9	p<0.001

Supplementary Table 2. Value of the elevation and the vegetation properties in the zones constituting the modeled salt marsh systems.

Zone	Elevation [m AMSL]	Vegetation Height [cm]	Vegetation Density [stems m^{-2}]	Stem Width [cm]
Marsh Platform	0.79	31.12	153.73	0.75
Levee Adjacent	0.79	60.36	124.47	1.30
Levees	0.94	89.59	95.20	1.85
Mussel Aggregations	0.79	63.27	136.80	1.37
Tidal Creek	0.79 → -1.00	0	0	0
Main Channel	-6.00	0	0	0

Supplementary Note 1

Converting Deposition to Accretion

The landscape assays of sediment deposition, i.e., the filter paper deployments, characterized the amount of sediment deposited in a discrete area (9-cm diameter filter paper, 63.6 cm^2). Previous work (3) has proposed the following equation for converting sediment deposition rate (SDR; g cm^{-2} yr^{-1}) into accretion rate (AR; cm yr^{-1}):

$$AR\ (cm\ year^{-1}) = SDR\ (g\ cm^{-2} year^{-1})/BD(g\ cm^{-3})$$

where BD is sediment bulk density in g cm^{-3}. We collected bulk density measurements from the region (4) and estimated an average bulk density to be between 0.3 (5) and 1 g cm^{-3} (3). We then compare measured filter paper deposition values of 0.02 (non-mound neap), 0.11 (non-mound spring), 0.08 (on mound neap) 0.39 (on mound spring), and 0.61 kg m^{-2} day^{-1} (summer on-mound spring) with values of accretion previously published from the region.

Our measured rates of accretion range from +0.01 cm yr^{-1} to +0.74 cm yr^{-1}, a range comparable to published accretion values reported in the region (-0.4 to +1.0 cm yr^{-1}; 6). We note that results from Experiment 2 were also in this same range, 0.04 - 0.40 kg m^{-2} day^{-1}, equivalent to (at the low end) 0.015 cm yr^{-1} and (at the high end) 0.487 cm yr^{-1}.

Supplementary Note 2

Creek Density Sensitivity Analysis

To test whether increasing the number of creeks within the creekshed would significantly alter the results from a single creekshed, we ran additional model iterations in which we considered a domain consisting of three tidal creeks and their creeksheds as one contiguous marsh domain. Each creek and creekshed has the geometry reported in Figure 5A, so the domain size is 150 m and 207 m in the long-shore and landward directions, respectively. We chose a density of mussel aggregation equal to 10% of the creekhead area, and the boundary conditions applied to the model were those described in the section "Methods - Delft3D Model". The max difference between deposition and accretion we computed for one creek in the 3-creek watershed (i.e., within one-third of the total 3-creek watershed) and the ones we computed from a single creek creekshed (and reported in the paper) is 2.5% of the original value. Given the small differences in results, and the already large scope of this work, we suggest that these results support our choice of using one single creek and creekshed throughout the paper.

Supplementary Note 3

Mussel Density Sensitivity Analysis

Mussel aggregations found at creekheads occur in a range of sizes (i.e., number of mussels they include). Large mounds with up to several hundred individuals typically occur closer to the creekhead, and they decrease in size with distance onto the marsh platform (Figure S2). To approximate these dynamics and to enable us to address our primary research question, we quantified an average creekhead mussel density representative of the entire 2,500m^2 creekhead area. To calculate this value, we surveyed 8 mussel aggregations at each of 3 distances from tidal creekheads (0m, 20m onto marsh platform, and 40m onto marsh platform) at ten creeks distributed across sites on Sapelo Island and the surrounding marshlands (n= 240 total mussel aggregations). We recorded total number of mussels in each aggregation as well as the mound dimensions. We then calculated the average mussel density per square meter and found this value to be 177 mussels m^{-2}. However, there is a great deal of variation, such that the standard deviation is 88 mussels.

To assess how sensitive the model results are to mussel density, we ran a new set of model iterations. We selected a scenario where mussels occupy 20% of the marsh area (to amplify their effects and potential differences across mussel density treatments). We then established three mussel density treatments, 89 mussels m^{-2} (mean – 1SD), 177 mussels m^{-2} (mean), and 265 mussels m^{-2} (mean + 1SD) and compared the sediment volume deposited in the whole domain in each scenario. We find that the effects of mussel density vary almost linearly with sediment volume contribution (and resulting accretion rate; Figure S9). Compared to the no-mussel scenario, the low mussel density scenario increases total sediment deposition in the domain by 1.33%, as compared with the intermediate density at 2.00%, and the high density by 2.55%. However, given that the mean value is representative of the average mound size, and that the relationship between mound size and contribution is linear, we use this value to provide an average estimate of their role in the greater creekhead area.

Supplementary References

1. S. Fagherazzi, M. Marani, and L. K. Blum. (2004). Introduction: The coupled evolution of geomorphological and ecosystem structures in salt marshes, in The Ecogeomorphology of Tidal Marshes, Coastal Estuarine Stud., vol. 59, edited by S. Fagherazzi, M. Marani,and L. K. Blum, pp. 1–5, AGU, Washington, D. C.
2. S. Crotty and C. Angelini (2020). Geomorphology and Species Interactions Control Facilitation Cascades in a Salt Marsh Ecosystem. *Current Biology*, **30**, 1562-1571.
3. C Butzeck, A Eschenbach, A Grongroft, and K Hansen. (2015). Sediment Deposition and Accretion Rates in Tidal Marshes Are Highly Variable Along Estuarine Salinity and Flooding Gradients. *Estuaries and Coasts*, **38**, 434-450.
4. PM Bradley and JT Morris. (1990). Physical characteristics of salt marsh sediments: ecological implications. *Marine Ecology Progress Series*, **61**, 245-252.
5. SC Neubauer, IC Anderson, JA Constantine, and SA Kuehl. (2002). Sediment deposition and accretion in a mid-Atlantic (U.S.A.) tidal freshwater marsh. *Estuarine, Coastal and Shelf Science* **54**, 713e727.
6. SM Crotty, *et al*. (2020). Sea-level rise and the emergence of a keystone grazer alter the geomorphic evolution and ecology of southeast US salt marshes. *Proc. Natl. Acad. Sci. U. S. A.* **117**, 17891-17902.
