# Supplementary material for: Faunal engineering stimulates landscape-scale accretion in southeastern US salt marshes
Source: Nat Commun. 2023 Feb 16;14:881. doi: 10.1038/s41467-023-36444-w (PMC9935860; doi:10.1038/s41467-023-36444-w)
Supplement: Supplementary file 3 — Reporting Summary [file 41467_2023_36444_MOESM3_ESM.pdf]

nature portfolio

Corresponding author(s): Sinead M. Crotty
Last updated by author(s): 01/19/2023

# Reporting Summary

Nature Portfolio wishes to improve the reproducibility of the work that we publish. This form provides structure for consistency and transparency in reporting. For further information on Nature Portfolio policies, see our Editorial Policies and the Editorial Policy Checklist.

## Statistics

For all statistical analyses, confirm that the following items are present in the figure legend, table legend, main text, or Methods section.

| n/a | Confirmed | |
|---|---|---|
| ☐ | ☒ | The exact sample size (*n*) for each experimental group/condition, given as a discrete number and unit of measurement |
| ☐ | ☒ | A statement on whether measurements were taken from distinct samples or whether the same sample was measured repeatedly |
| ☐ | ☒ | The statistical test(s) used AND whether they are one- or two-sided<br>*Only common tests should be described solely by name; describe more complex techniques in the Methods section.* |
| ☐ | ☒ | A description of all covariates tested |
| ☐ | ☒ | A description of any assumptions or corrections, such as tests of normality and adjustment for multiple comparisons |
| ☐ | ☒ | A full description of the statistical parameters including central tendency (e.g. means) or other basic estimates (e.g. regression coefficient) AND variation (e.g. standard deviation) or associated estimates of uncertainty (e.g. confidence intervals) |
| ☐ | ☒ | For null hypothesis testing, the test statistic (e.g. *F*, *t*, *r*) with confidence intervals, effect sizes, degrees of freedom and *P* value noted<br>*Give P values as exact values whenever suitable.* |
| ☒ | ☐ | For Bayesian analysis, information on the choice of priors and Markov chain Monte Carlo settings |
| ☒ | ☐ | For hierarchical and complex designs, identification of the appropriate level for tests and full reporting of outcomes |
| ☒ | ☐ | Estimates of effect sizes (e.g. Cohen's *d*, Pearson's *r*), indicating how they were calculated |

*Our web collection on statistics for biologists contains articles on many of the points above.*

## Software and code

Policy information about availability of computer code

| | |
|---|---|
| Data collection | No software was used for data collection. |
| Data analysis | We used R version 3.1.0 for regression tree analysis and STATA v 15.1 for simple parametric analyses. To generate the Delft3D-BIVALVES model, we utilized the Delft3D-FLOW model (https://oss.deltares.nl/web/delft3d). Finally, we used CloudCompare software version 2.12.4 for creation of our 2020 DEM (https://github.com/cloudcompare/cloudcompare). |

For manuscripts utilizing custom algorithms or software that are central to the research but not yet described in published literature, software must be made available to editors and reviewers. We strongly encourage code deposition in a community repository (e.g. GitHub). See the Nature Portfolio guidelines for submitting code & software for further information.

## Data

Policy information about availability of data

All manuscripts must include a data availability statement. This statement should provide the following information, where applicable:
- Accession codes, unique identifiers, or web links for publicly available datasets
- A description of any restrictions on data availability
- For clinical datasets or third party data, please ensure that the statement adheres to our policy

The data generated in this study have been deposited in the Figshare database under accession code: https://doi.org/10.6084/m9.figshare.13177100.v2.

## Human research participants

Policy information about studies involving human research participants and Sex and Gender in Research.

| | |
|---|---|
| Reporting on sex and gender | N/A |
| Population characteristics | N/A |
| Recruitment | N/A |
| Ethics oversight | N/A |

Note that full information on the approval of the study protocol must also be provided in the manuscript.

# Field-specific reporting

Please select the one below that is the best fit for your research. If you are not sure, read the appropriate sections before making your selection.

☐ Life sciences ☐ Behavioural & social sciences ☒ Ecological, evolutionary & environmental sciences

For a reference copy of the document with all sections, see nature.com/documents/nr-reporting-summary-flat.pdf

# Ecological, evolutionary & environmental sciences study design

All studies must disclose on these points even when the disclosure is negative.

Study description

Landscape Assays of Sediment Deposition over Seasons and Tidal Phases
Description: The landscape assays of sediment deposition involved two sets of filter paper deployment:
1) 9-cm filter paper deployed across 13 location types and 4 tides (minimum 15 replicates each)
2) 4.7-cm filter paper deployed across 5 location types and 1 tide and analyzed for organic and inorganic C content (5 replicates each)

Design Structure: Marsh location types were selected to represent landscape maxima and minima of deposition according to the literature and our hypotheses; For the first filter deployment, the 4 tides were selected to represent a range of submersion time (i.e., spring vs. neap) and temperature (i.e., summer vs. winter); Factorial (every location is replicated in every tide).

Treatment factors and interactions: Location Type, Tide, Location*Tide

Nature and Number of experimental units and replicates:
For the first deployment (4 tides), we deployed a minimum of 15 replicate filters at each of 13 locations (Whatman Quantitative Filter Paper, Grade 42 Circles, Ashless, 90 mm).
For the second deployment (1 tide), we deployed 5 replicate filters at each of 5 location types (Whatman Glass Microfiber Filter Paper, Grade GF/F Circles, 47 mm).

Field Experiment 1: Fate of Mussel Biodeposits
Description: The fate of mussel biodeposits were assessed with two methods: 1) transport of previously settled biodeposits and 2) transport of actively ejected biodeposits over 1 tidal cycle.

Design Structure:
For previously settled biodeposits, 6 mussel mounds were selected in each of 2 zones. Experimental zones were 1) tidal creekhead and 2) 20-m away from the creekhead on the higher-elevation marsh platform. Experimental mounds were separated by >5m to avoid mixing of tagged biodeposits. For each mound, we removed 2cm of each mound’s biodeposit layer, homogenized it with fluorescent chalk (Irwin Straight-Line Fluorescent Orange Marking Chalk), redistributed it back on the mound, and then revisited the mounds at night after one tide had flooded over the mounds. The distribution of fluorescent material was traced through black light detection in N, S, E, and W directions. There were no differences in distance traveled in any direction, so we instead present results of maximum distance traveled per tide in each zone.

For actively ejected biodeposits, we collected 10 mussels from each mound, depurated them in saltwater (Instant Ocean, 28 ppt) for 24 hours, and allowed them to feed on a mixture of seawater and fluorescent chalk for 2 hours. We then transplanted them back into the focal mounds at low tide. We then revisited the mounds at night after one tide had flooded over the mounds and traced the distribution of fluorescent material through black light detection. detection in N, S, E, and W directions. There were no differences in distance traveled in any direction, so we instead present results of maximum distance traveled per tide in each zone.

Treatment factors and interactions: Zone, Direction, Zone*Direction (only report results of main effect zone as described above)

Nature and Number of experimental units and replicates: For previously settled biodeposits, we used 6 replicate mounds in each of two zones (12 total replicates). For actively ejected biodeposits, we used 10 mussels per mound, 6 replicate mounds, and two zones (12 total mounds, 120 total mussels utilized for measurement).

Field Experiment 2: Local Scale Depositional Effects of Mussels and Cordgrass
Description: Local scale depositional effects of mussels and cordgrass were assessed through a fully-factorial field experiment consisting of 7 experimental treatments replicated across 2 marsh zones.

Design Structure: The experiment was deployed at two locations at a site on Sapelo Island: one high elevation marsh platform >85m from the nearest tidal creek (31°25'25.3"N 81°17'29.8"W) and one lower elevation creekhead (31°25'28.1"N 81°17'30.2"W). Within each marsh location, we deployed seven experimental treatments (N = 5 replicates per treatment per location), including: 1) no-mussel, no-cordgrass controls; 2) cordgrass-only controls; 3) 1-mussel (no cordgrass) blocks; 4) small mussel aggregations (20 mussels, no-cordgrass); 5) intermediate size mussel aggregations (50 mussels, no cordgrass); 6) intermediate size mussel aggregations plus cordgrass (50 mussels); and 7) large mussel aggregations (80 mussels, no cordgrass; Figure S3).

Treatment factors and interactions: In each zone, we used a multiple regression analysis with cordgrass biomass and mussel biomass as predictor variables for deposition. We elected to used continuous predictors (i.e., mussel biomass and cordgrass biomass) rather than categorical variables (i.e., Treatment) because both mound size and cordgrass biomass varied within each treatment; this approach allowed for incorporation of that variation.

Nature and Number of experimental units and replicates: See Figure S3 for nature of experimental units. We deployed 5 replicates of each treatment (7 total) in each zone (2 total), for a total of 70 replicates.

Field Experiment 3: Manipulation of Landscape Mussel Populations
Description: Approximately 200,000 mussels were removed from one tidal creekhead and transplanted to another creekhead in summer 2017 (full manipulation plot size: 10,000 m2; creekhead plot size: 2,500 m2). These experimental plots as well as one un-manipulated control plot of the same size were assessed for changes to elevation over a 3-year period.

Design Structure: Due to logistical and permitting constraints, it was not feasible to replicate the treatments across multiple sites; instead, the three plots occupied a single contiguous creekshed (Figure 4A-B). Initial elevation was assessed with two methods: Real Time Kinematic (RTK) elevation datapoints (minimum of 20 per creekhead) and mussel height ceiling calculations. Final elevation was assessed using a Digital Elevation Model (DEM). We assess the differences between creekhead elevation of the experimental treatments and the unmanipulated control at each time point and for each method.

Treatment factors and interactions: Treatment (i.e., control, removal, addition)

Nature and Number of experimental units and replicates: The experiment was deployed only at one site (as described above). However, within each plot, we assessed initial elevation with a total of 86 RTK points (20 minimum per creekhead) and 250 mussel mound heights (>60 per creekhead). Final elevation was summarized across the entire 2,500m2 creekhead area from DEM data.

Research sample

Landscape Assays of Sediment Deposition over Seasons and Tidal Phases
Research Sample: N/A

Field Experiment 1: Fate of Mussel Biodeposits
Research Sample: 120 individuals of Geukensia demissa were removed from field aggregations and transported to University of Georgia Marine Institute’s wet lab, depurated in saltwater (Instant Ocean, 28 ppt) for 24 hours, and then fed a mixture of seawater and fluorescent chalk for 2 hours. The 120 individuals were randomly selected from across 12 aggregations. They ranged in size from 4-10cm in length and were distributed back to mounds such that all mounds had similar size distributions of individuals (3 individuals 4-6cm; 4 individuals 6-8cm; 3 individuals 8-10cm). The size distribution utilized directly mimicked the natural size distribution present in Sapelo Island marshes (see Crotty & Angelini 2020 for greater detail on mussel size distribution). The research sample represents the population of ribbed mussels in creekhead and marsh platform locations on Sapelo Island.

Field Experiment 2: Local Scale Depositional Effects of Mussels and Cordgrass
Research Sample: For research samples, 10 blocks containing small natural Geukensia demissa aggregations (~20 mussels), 20 blocks of intermediate-size Geukensia demissa aggregations (~50 mussels), and 10 blocks of large Geukensia demissa aggregations (~80 mussels). Experimental blocks were selected at the experimental site based on surficial counts of mussels (20-80 individuals). For the 1-mussel treatments, we harvested 10 Geukensia demissa individuals (6-8cm in length) from the experimental site and individually inserted them in the center of the marsh block so that they were 40-50% below the marsh surface. All mounds and individuals were selected from representative creekhead areas, and are reflective of mussel populations in the region.

Field Experiment 3: Manipulation of Landscape Mussel Populations
Research Sample: N/A

Sampling strategy

No sample size calculation was completed. All decisions for sample size and replication were selected based on field and permitting constraints. The sampling procedure for field experiment 1 involved removing individual mussels from 12 representative mussel aggregations from across a range of marsh location types between the creekhead and the marsh platform. In field experiment 2, the sampling procedure involved visiting >100 mounds, scoring the total number of mussels per mound, and selecting those mounds where the number of individuals was closest to the groups included in the experiment (20, 50, and 80).

Data collection

Landscape Assays of Sediment Deposition over Seasons and Tidal Phases: Sinead Crotty deployed all filter papers and recorded all data from measurements directly into digital form. Data includes measurement of filter paper initial weight, aluminum packet initial weight, combined dry weight (filter plus sediment plus aluminum packet), and calculated weight of sediment.

Field Experiment 1: Fate of Mussel Biodeposits: Sydney Williams and Hallie Fischman deployed both phases of Experiment 1, traced the distribution of fluorescent material through black light detection (ultilizing a compass for cardinal direction and a transect tape for distance measurement), and recorded all data in the field. Data was then transcribed into digital form. Data includes measurement of maximum distance sediment traveled from each mound in each marsh zone.

Field Experiment 2: Local Scale Depositional Effects of Mussels and Cordgrass: Sinead Crotty harvested the full experiment. Sediment was collected from the sediment capture devices in a few forms. First, all liquid was bilged out of the catchment unit and into labelled 1-gallon jugs. Next, all sediment was removed and placed into pre-weighed, pre-labelled aluminum tins. Once back at the laboratory, all surficial sediment was removed from central mussel aggregations using spatulas, scraper tools, and a Waterpik Flosser device. All sediment was then dried at 70C in aluminum tins (including liquid which was redistributed across many tins) and weighed. Data was entered directly into digital form. Cordgrass biomass was similarly dried, weighed, and entered into digital form.

Field Experiment 3: Manipulation of Landscape Mussel Populations: Initial data was collected in the field by Sinead Crotty and Hallie Fischman. Transects were established 50m in length using a transect tape oriented perpendicular to the creek point of entry, with its center point at the creekhead. Three transects were conducted (0m from creekhead, 20m, and 40m). Each transect was 1m wide. Mounds were included if >50% of their area was inside the 50m2 transect. All survey measurements were assessed by Sinead Crotty and transcribed by Hallie Fischman, who subsequently entered them in digital form.

To assess elevation in 2020, we compared creekhead elevation using a 2020 Digital Elevation Model (DEM) of the creekshed. To build the DEM, we flew a DJI Matrice 600 Pro drone carrying a custom build Lidar payload in August 2020 (led by Andrew Ortega). The payload consisted of a Velodyne Puck Lite VLP16, paired with a Novatel Stim300 Inertial Measurement Unit. The point clouds from the drone were orthorectified from GPS data continuously measured on the drone (see the procedure described in 68, 69). To remove the vegetation and any other surface perturbations (i.e., from digital surface model to digital elevation model), we used the CloudCompare software (https://github.com/cloudcompare/cloudcompare). The cloth Simulation Filter (CSF; 70) was applied twice to the dataset, which successfully removed the vegetation data. The point cloud of the marsh surface was then exported to ArcGIS 10.7 where the DEM was generated by raster interpolation. Once completed, the mean elevation within each 2,500m2 creekhead location was calculated using the Zonal Statistics tool in ArcGIS 10.7 by Collin Ortals.

Timing and spatial scale

Landscape Assays of Sediment Deposition over Seasons and Tidal Phases: In each deployment, filter papers (9-cm and 4.7-cm diameter) were left in the field over one tidal cycle (deployed at low tide, submerged at high tide, harvested at low tide). We limit the deployment time to one tidal cycle to understand the amount deposited over short time scales and to avoid loss to rain or other factors that affect assessment of rates (e.g., bioturbation). We then additionally deployed 9-cm filters over 4 tidal cycles (summer neap, summer spring, winter neap, winter spring) to assess the variation in deposition on this time scale in different tidal and seasonal conditions.

Field Experiment 1: Fate of Mussel Biodeposits: We assess redistribution over one tidal cycle. We limit this study to this short time period so that we don’t risk the mixing of material across mounds.

Field Experiment 2: Local Scale Depositional Effects of Mussels and Cordgrass: We deployed this experiment for one month. All data was collected at the harvest time point. The spatial scale is ~1-m2, and is compared against the filter paper results (deposition on 9-cm diameter devices).

Field Experiment 3: Manipulation of Landscape Mussel Populations: This experiment was deployed in the Summer of 2017. All initial data was collected prior to initiating treatment effects. Sampling and site visitation was minimal to reduce effects of trampling. End point data was collected for 2,500m2 areas using a Digital Elevation Model (DEM) after 3 years.

Data exclusions

Landscape Assays of Sediment Deposition over Seasons and Tidal Phases: For Part 1, we exclude filter deployment locations in close proximity to "grazed" creekheads, characterized by burrowed, sunken tidal creekheads, low vegetation cover, and no mussel aggregations. We initially took filter paper measurements at a larger number of locations (termed "low muck" "high muck" "0m grazed" and so on), but removed a subset from the analysis because they represent a minority of creekheads and were not relevant to our current study. See Crotty et al. 2020 (https://www.pnas.org/doi/abs/10.1073/pnas.1917869117) for more information on these area types.
Field Experiment 1: Fate of Mussel Biodeposits: No data were excluded from the analysis.
Field Experiment 2: Local Scale Depositional Effects of Mussels and Cordgrass: No data were excluded from the analysis.
Field Experiment 3: Manipulation of Landscape Mussel Populations: No data were excluded from the analysis.

Reproducibility

Reproducibility was addressed by deploying experiments or assays across a range of tides, sites, elevational zones, etc. In addition, all small scale experiments and assays included a minimum of 6 replicates (and some with >60 replicates). We confirm that (with the exception of the filter paper location types excluded) all replicates were successful. The large scale mussel manipulation experiment was only replicated at one site owing to permitting and personnel constraints. We acknowledge this limitation in the manuscript.

Randomization

In all cases where individuals were assigned to groups (e.g., the mussel individuals harvested and replanted in Experiment 1), we first collected a representative sample of mussel individuals. Mussels were separated into 6 size classes and exposed to the experimental treatment. They were then deployed in two zones: the creekhead and the marsh platform. For deployment in each zone, one mussel of each size class was randomly selected and then subsequently deployed (for a total of 6 replicates per zone).

Blinding

Blinding was not possible for much of the data collection and analysis because different treatments have visually apparent variations. However, all initial data collection for the large scale mussel manipulation, for example, was recorded prior to randomly assigning each creekhead into a treatment. In addition, all filters and aluminum tins were numbered such that the recorder would have no knowledge of the location of deployment in the marsh or within a certain treatment without the code key.

Did the study involve field work? ☒ Yes ☐ No

## Field work, collection and transport

| | |
|---|---|
| Field conditions | Sapelo Island, located between latitudes of 31° 32.3' and 31° 22.8' N, is a barrier island on the Georgia coast (19 kilometers long and 5 km wide). The climate on Sapelo is temperate to subtropical; temperatures range from a mean high of 90°F in the summer to 50°F in the winter, and rarely goes below freezing. Rainfall is about 50 inches/year, with most precipitation during hurricane season (May-Sept). Field work was conducted throughout the majority of low tides in the spring & summer of 2017 (early May through late Sept for all deployment, harvesting, data collection, and recording), and additionally included two tidal cycles in Feb 2018.<br><br>Additional field data was collected in the summer months of 2020 (May - August). Temperature and precipitation were similar across the years of study. |
| Location | Landscape Assays of Sediment Deposition over Seasons and Tidal Phases: Performed on marshes on mainland Sapelo (Google Earth file of exact locations available upon request).<br><br>Field Experiment 1: Fate of Mussel Biodeposits: The first experimental study was conducted at Beach Road marsh on Sapelo Island, Georgia, USA (31°23'26.0"N 81°16'26.0"W).<br><br>Field Experiment 2: Local Scale Depositional Effects of Mussels and Cordgrass: The second experimental study was conducted at Airport Marsh on Sapelo Island, Georgia, USA. At this site, the experiment was deployed at two locations: one high elevation marsh platform >85m from the nearest tidal creek (31°25'25.3"N 81°17'29.8"W) and one lower elevation creek head, where the tidal creek enters onto the marsh platform and tidal water first enters the marsh platform (31°25'28.1"N 81°17'30.2"W).<br><br>Field Experiment 3: Manipulation of Landscape Mussel Populations: The third experimental study was conducted at a marsh island in close proximity to Sapelo Island. The mussel removal creek can be found at these coordinates: 31°23'26.0"N 81°16'26.0"W. The mussel addition creek can be found at these coordinates: 31°23'26.0"N 81°16'26.0"W. The unmanipulated control creek can be found at these coordinates: 31°23'26.0"N 81°16'26.0"W. |
| Access & import/export | All field deployments were permitted by both the Georgia Coastal Ecosystems LTER as well as the Georgia Department of Natural Resources. GCE-LTER permits can be found here: https://gce-lter.marsci.uga.edu/public/app/view_requests.asp. Relevant permits for this manuscript are: GCE-84-2017 and GCE-86-2017. For GA DNR, we have authorizations from the Coastal Resources Division (LOP20170162 and LOP20170167). Our scientific collections are covered under Master Permit # 029-1000531205. |
| Disturbance | Minimizing disturbance was key to the success of our experimental deployments, as movement of sediment was what we aimed to capture. Thus, in all instances, the number of field personnel was limited to those necessary to complete work. All physical steps were carefully executed such that cordgrass was not trampled and damaging trails were not established and maintained. For all movement of heavy blocks of sediment or large number of mussels, sleds were utilized at high tide (or on rising tides) to aid in movement and to minimize disturbance to the site. For excavation of sediment to allow for insertion of sediment catchment devices, all shoveling/excavation was carefully conducted from within the plot boundary such that no edges were trampled. For the large scale mussel manipulation, it was not possible to completely exclude the use of trails because of the large extent of the manipulation. However, we limited the establishment of trails to areas outside the creekhead area (2500m2). These trails are visible in aerial imagery in 2018 but are no longer present by 2020, suggesting that the disturbance was minimal. |

# Reporting for specific materials, systems and methods

We require information from authors about some types of materials, experimental systems and methods used in many studies. Here, indicate whether each material, system or method listed is relevant to your study. If you are not sure if a list item applies to your research, read the appropriate section before selecting a response.

## Materials & experimental systems

| n/a | Involved in the study | |
|---|---|---|
| ☒ | ☐ | Antibodies |
| ☒ | ☐ | Eukaryotic cell lines |
| ☒ | ☐ | Palaeontology and archaeology |
| ☐ | ☒ | Animals and other organisms |
| ☒ | ☐ | Clinical data |
| ☒ | ☐ | Dual use research of concern |

## Methods

| n/a | Involved in the study | |
|---|---|---|
| ☒ | ☐ | ChIP-seq |
| ☒ | ☐ | Flow cytometry |
| ☒ | ☐ | MRI-based neuroimaging |

## Animals and other research organisms

Policy information about studies involving animals; ARRIVE guidelines recommended for reporting animal research, and Sex and Gender in Research

| | |
|---|---|
| Laboratory animals | This study did not involve laboratory animals. |
| Wild animals | See section below on field-collected samples. |
| Reporting on sex | Sex was not considered in study design. |
| Field-collected samples | Geukensia demissa individuals and mounds were collected from the field for several parts of this study. For Experiment 1, 120 mussel individuals were harvested from across 12 mounds at one site. Care was taken to ensure no damage to the byssus. The mussels were moved to the University of Georgia Marine Institute's wet lab, depurated in saltwater (Instant Ocean, 28 ppt) for 24 hours, and allowed to feed on a mixture of ambient seawater and fluorescent chalk for 2 hours. We then rinsed the mussels to remove any loose fluorescent material from their shells before transplanting them back during the next low tide into the focal mounds.<br><br>For Experiment 2, natural mussel aggregations were harvested from the field, cleaned, and transplanted into sediment catchment devices. Harvested aggregations were housed in University of Georgia Marine Institute's wet lab for approximately 72 hours. During this process, to reduce new biodeposits after cleaning, mussel aggregations were not exposed to tidal inundation. Temperature of the lab setting was approximate 75F, and experimental units were returned to the field as soon as possible. At the end of the experimental deployment, sediment catchment devices and mussels contained within them were harvested and returned to the lab for analysis. Mounds were carefully dismantled, and each mussel was measured for length and weighed. After measurement, mussels were returned to the experimental site and replanted in mounds near to where they were initially harvested. |
| Ethics oversight | No ethical approval was required for the organisms of interest in the study. All proper permits were acquired for conducting the work. |

Note that full information on the approval of the study protocol must also be provided in the manuscript.
